# Traveling Does More Than Widen One’s Horizon: Travel History as Key to Diagnosing Blastomycosis

**DOI:** 10.7759/cureus.38551

**Published:** 2023-05-04

**Authors:** Ana M Alvarez, Melanie Vega, Mobeen Rathore

**Affiliations:** 1 Pediatrics/Infectious Diseases, University of Florida College of Medicine, Jacksonville, USA

**Keywords:** non-resolving pneumonia, necrotizing pneumonia, fungal pneumonia, travel history, pulmonary blastomycosis

## Abstract

This case report highlights the importance of a detailed travel history and the need to revisit the differential diagnosis when there is an unexpected clinical course. A previously healthy 15-year-old male presented to a hospital in Florida with a fever, cough, and shortness of breath. He was seen multiple times at urgent care centers and treated with steroids and antibiotics for community-acquired pneumonia (CAP). The patient’s chest X-rays and CT showed necrotizing pneumonia with pleural effusion, which required a chest tube. Despite broadening coverage for possible resistant organisms, his fevers and hypoxia continued. On day 14 of hospitalization, a bronchoscopy was performed, which led to the diagnosis of blastomycosis. History was revisited, and a specific travel history was obtained. The patient had been camping with his father on the Minnesota/Canada border a few months prior to his presentation.

Blastomycosis is caused by a dimorphic fungus endemic in certain parts of the United States including areas surrounding the Mississippi and Ohio River valleys, some southeastern states, and areas bordering the Great Lakes. Autochthonous blastomycosis is not seen in Florida. The infection is acquired by inhalation of the organism and is associated with outdoor occupation and recreation. As with other infections with specific endemic distribution, the diagnosis of blastomycosis can be delayed if the epidemiologic link is not established. Questions about travel history need to be very specific as this could be critical in establishing the appropriate differential diagnosis and leading the workup. The patient’s lack of improvement despite appropriate antibiotic therapy for CAP led to questioning the working diagnosis, revisiting the history, and expanding the workup, which was critical in this case.

## Introduction

A thorough history and a focused physical exam are the bases for formulating differential diagnoses. For infectious diseases, it is especially important that the history includes exposures to sick people, animals, foods, specific environments, occupations, hobbies, and travel. The travel history, in particular, should be very specific in regard to the place, duration, activities, exposures, and timing in relation to the beginning of symptoms. In this case report, a patient with what initially seemed like uncomplicated community-acquired pneumonia (CAP) failed to respond to treatment of common organisms such as *Streptococcus pneumoniae*, *Mycoplasma pneumonia*, and resistant ones like methicillin-resistant *Staphylococcus aureus*. His clinical course never followed the typical course for those infections. On admission, he and his parents denied any travel history. However, on revisiting the travel history, they recalled he had traveled to areas where other less common causes of pneumonia are endemic. This case of pulmonary blastomycosis diagnosed in a non-endemic area highlights the importance of a detailed travel history and the need to revisit the differential diagnosis when there is an unexpected clinical course.

## Case presentation

**Figure 1:**
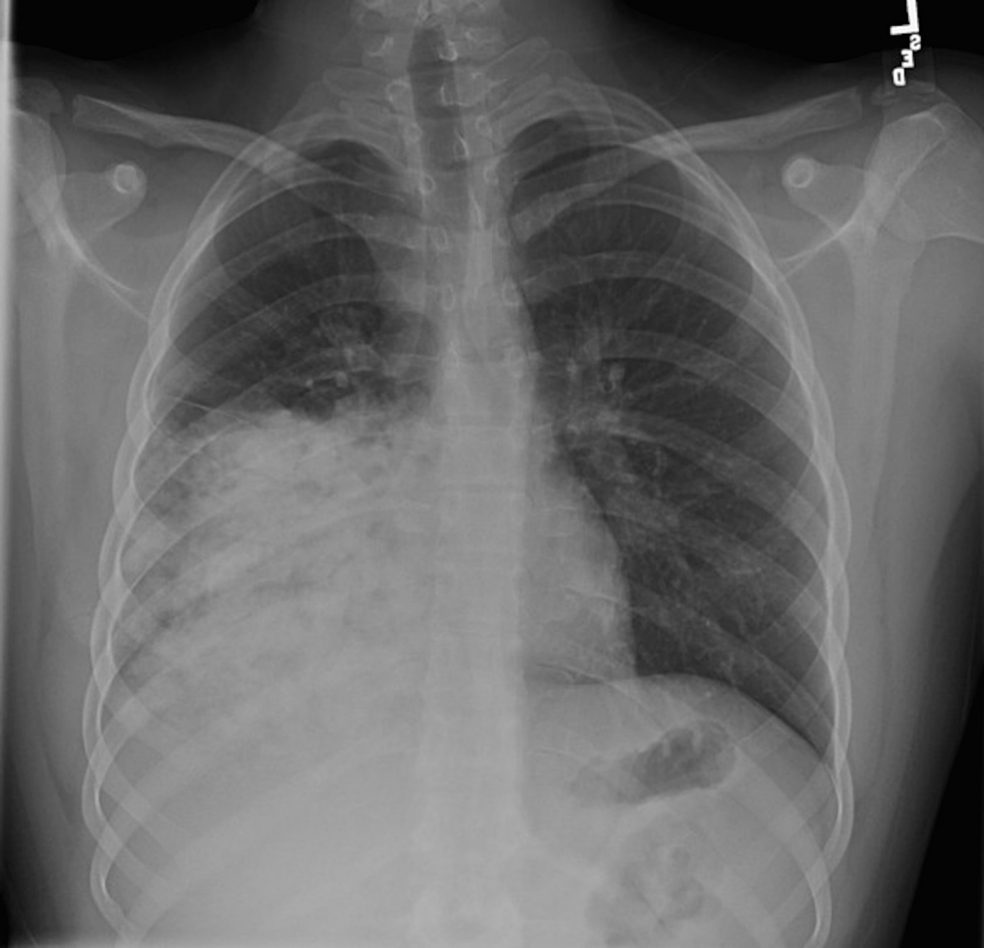
Chest X-rays on admission There was dense heterogeneous confluent opacity throughout the right middle and right lower lobe partially obscuring the right cardiac border. Trace right pleural fluid was observed.

A previously healthy 15-year-old male presented to a hospital in Florida with fever, cough, and shortness of breath. Four weeks earlier, he started with cold-like symptoms. Since then, he was seen four times in acute care settings, and he was diagnosed with an upper respiratory infection (URI) and, subsequently, with CAP based on chest radiographs (CXR). He had received treatment with cough medicine, steroids, and multiple courses of antibiotics, including oral azithromycin, oral cefdinir, and IV ceftriaxone. On the day of admission, he had a worsening dry cough, shortness of breath, and a fever of 104.7° F. He required 3 L/min of oxygen per nasal cannula to maintain his oxygen saturation in the mid-90s. CXR showed a dense opacity throughout the right middle and lower lobes with a small pleural effusion consistent with CAP (Figure 1).

Past medical history was unremarkable, except for environmental allergies. He and his parents denied a history of recurrent infections, travel, or exposure to sick contacts. Animal contact included four cats and one dog. Immunizations were up to date.

Admission vital signs were temperature 99.7° F, blood pressure 118/85 mm of Hg, and pulse rate 120/minute. The patient appeared mildly distressed with increased work of breathing. Auscultation on the left was clear. However, on the right side, there was diminished air movement throughout and inspiratory crackles on the lower lobe. The rest of his examination was unremarkable. White blood cell count was 15,800/mm3 with 81% segmented neutrophils, 11% lymphocytes, 7% monocytes, and 1% eosinophils. Platelet count was 336,000/mm3, hemoglobin 13.9/dL, and hematocrit 40.6%. C-reactive protein was 49.1 mg/dL and ESR 111 mm/hr. A complete metabolic panel, including renal and liver function tests, was normal. Two sets of blood cultures were obtained. SARS-CoV-2 and influenza molecular tests were negative.

**Figure 2:**
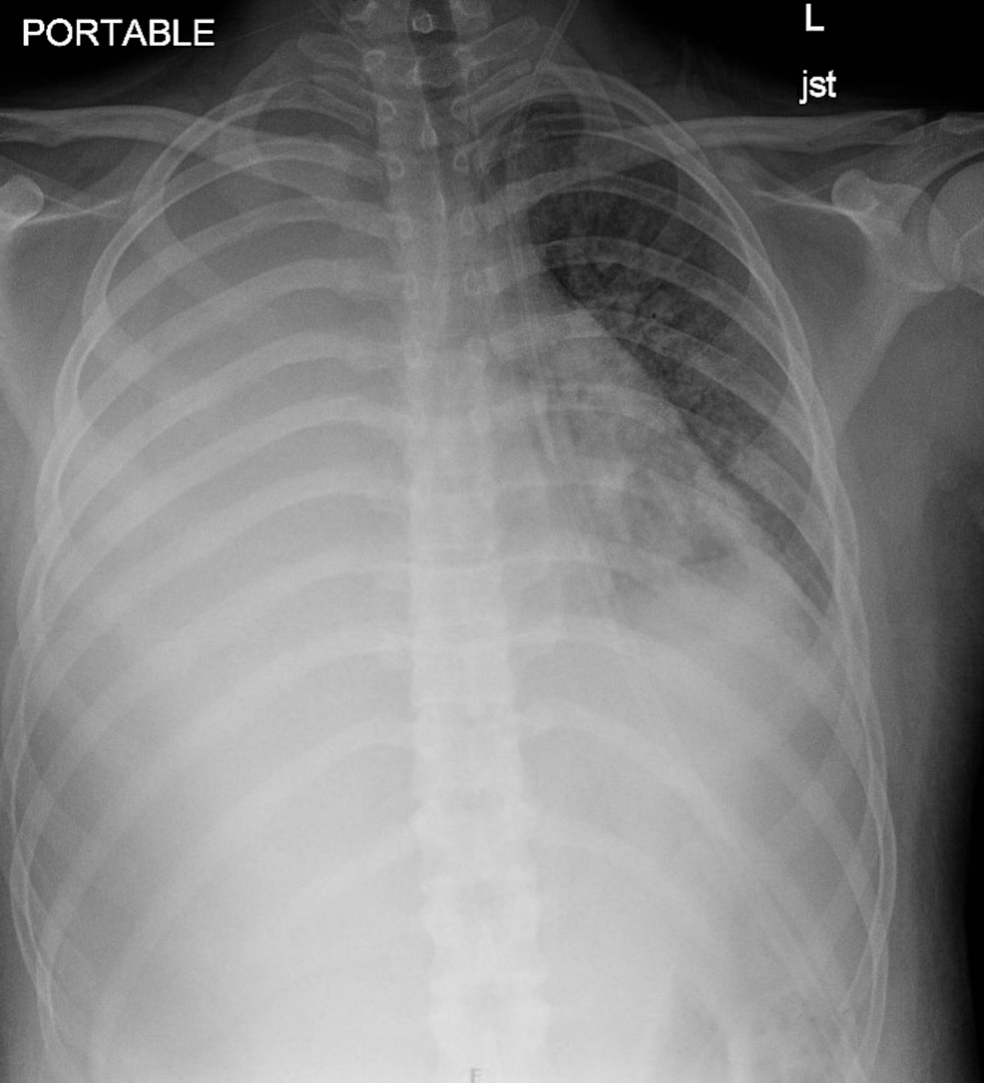
Chest X-ray on day five of hospitalization Complete opacification of the right lung and large right pleural fluid was observed. There were new interstitial and patchy airspace opacities in the left perihilar region and lung base.

He was admitted and treated with intravenous ceftriaxone and clindamycin. Over the next several days, he had remittent fevers, ongoing respiratory distress, and poor oral intake. Blood cultures remained sterile. Clindamycin was switched to linezolid to optimize gram-positive coverage. His symptoms persisted, and a repeat CXR on day five of hospitalization showed worsening opacities and a large pleural effusion (Figure 2).

**Figure 3:**
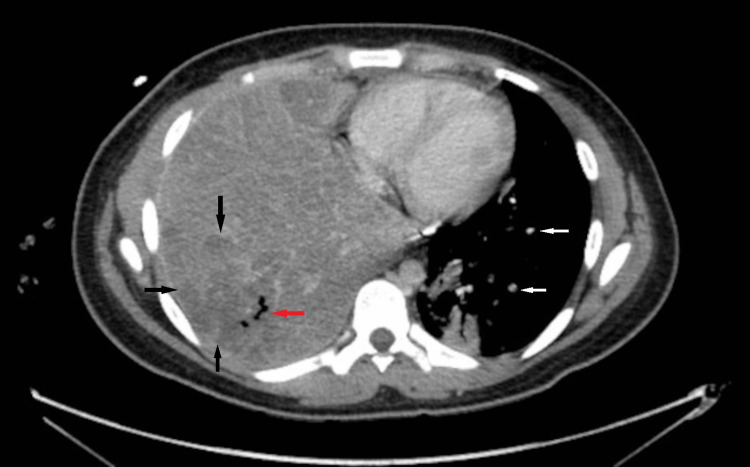
CT of the chest There was complete consolidation of the right middle and lower lobes with subtotal consolidation of the right upper lobe. Multifocal areas of diminished parenchymal enhancement (black arrows) and a few small areas of parenchymal cavitation (red arrow) in the right middle and lower lobes were observed. There were nodular opacities in the left lower lobe (white arrows).

A chest tube was placed and 550 ml of clear yellowish fluid was collected and sent for bacterial and fungal cultures. After a few days, the patient continued having fevers, and a chest CT was done. It showed right-sided consolidation with small cavitations consistent with necrotizing pneumonia (Figure 3).

Bacterial cultures from pleural fluid were negative. QuantiFERON TB Gold was indeterminate twice, and the tuberculin skin test was negative (0 mm of induration). Antibiotics were changed to ceftaroline and levofloxacin to cover possible multi-drug-resistant organisms.

**Figure 4:**
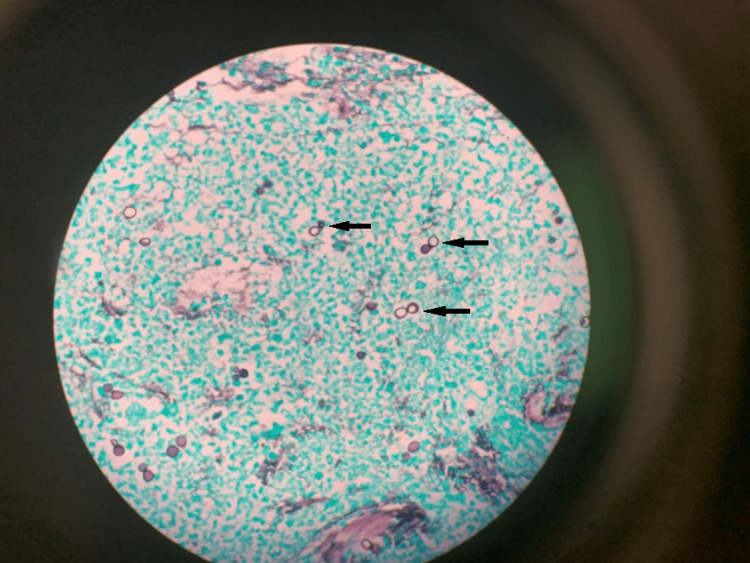
BAL cytology The specimen was cellular with numerous thick-walled budding yeast forms, neutrophils, alveolar macrophages, and a few erythrocytes. There were multiple fungal organisms (positive GMS stain), containing thick cell walls with a single rounded base (black arrows pointing out a few of many organisms seen). No hyphae were seen. Morphologically, the organisms resembled Blastomyces species.

Due to unrelenting fever and hypoxia after 14 days of hospitalization, the patient underwent bronchoscopy with bronchoalveolar lavage (BAL). Findings included mucosal edema with white plaques in his airways, which supported the possibility of a fungal infection. The bacterial cultures were negative. The acid-fast bacilli stain was negative. Calcofluor stain showed budding yeast and the fungal culture grew yeast several days later. The isolate was sent to a reference laboratory for identification. The BAL cytology demonstrated numerous thick-walled budding yeast forms, and it was positive for fungal organisms using Grocott-Gomori’s methenamine silver (GMS) stain. These findings were consistent with blastomycosis (Figure 4).

The family was questioned one more time, and they recalled camping trips to the Minnesota/Canada border and to North Carolina, two to three months and two to three weeks before the onset of symptoms, respectively.

**Figure 5:**
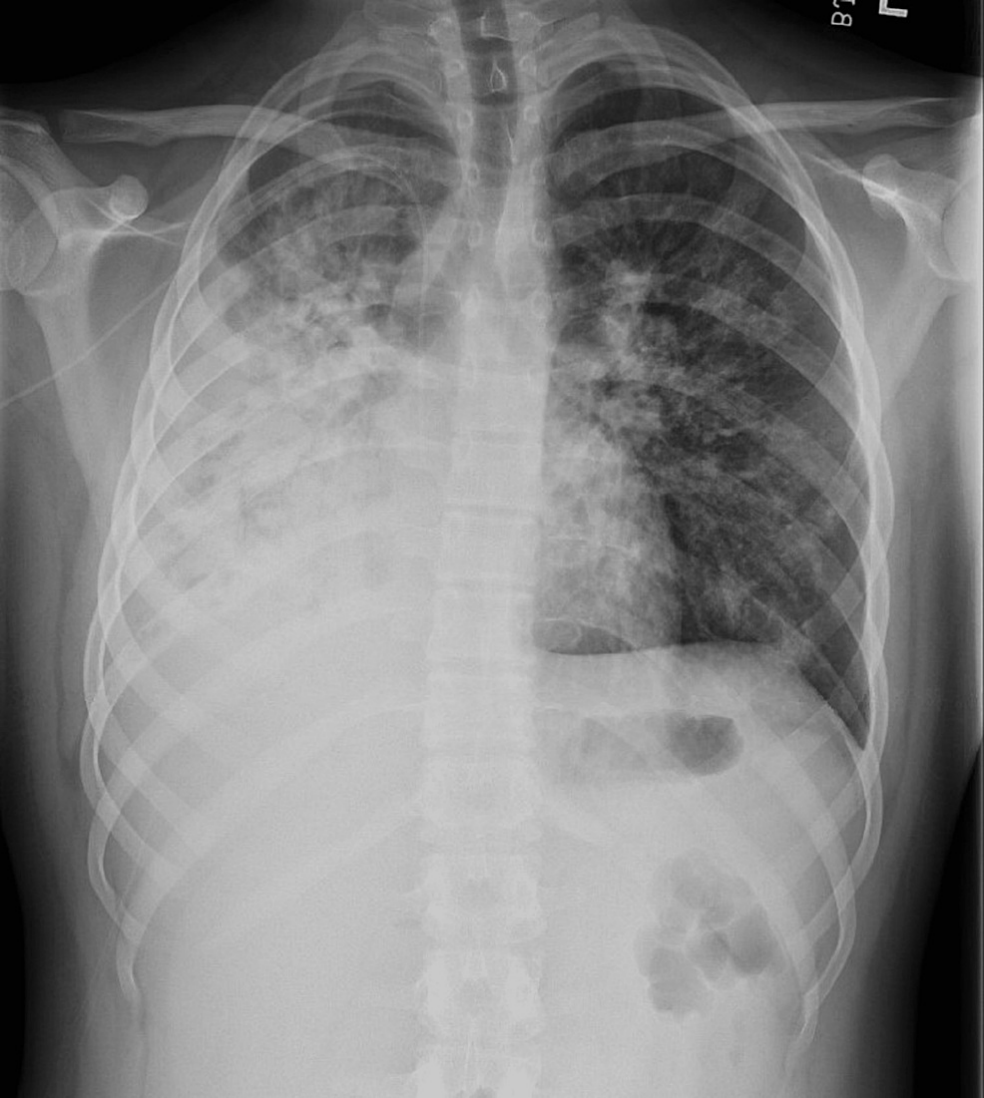
Chest X-rays after two weeks of treatment Extensive airspace disease was seen within the right mid and lower lung zone. There was minimally improved aeration in the right lower lobe. Right pleural effusion was identified as well. Patchy airspace disease was seen throughout the left mid and lower lung zone as well.

Our patient was started on liposomal amphotericin B. He continued to have daily fevers and be intermittently hypoxic for about one week into appropriate treatment. After two weeks, his CXR showed mild improvement (Figure 5), and he no longer required oxygen and was able to walk without getting short of breath, and his appetite started improving.

**Figure 6:**
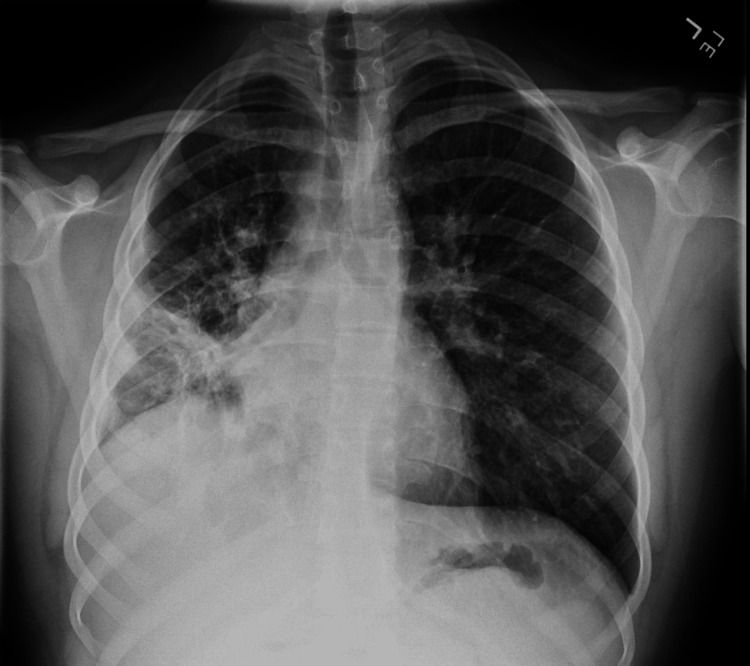
Chest X-rays after two months of treatment Right lung aeration appeared improved. Perihilar and right lower lobe coarsened interstitial markings and multifocal streaky parenchymal opacities were seen. Right-sided pleural fluid or thickening is identified. Left lung was well inflated with some persistent coarsening of interstitial markings and faint patchy alveolar densities.

**Figure 7:**
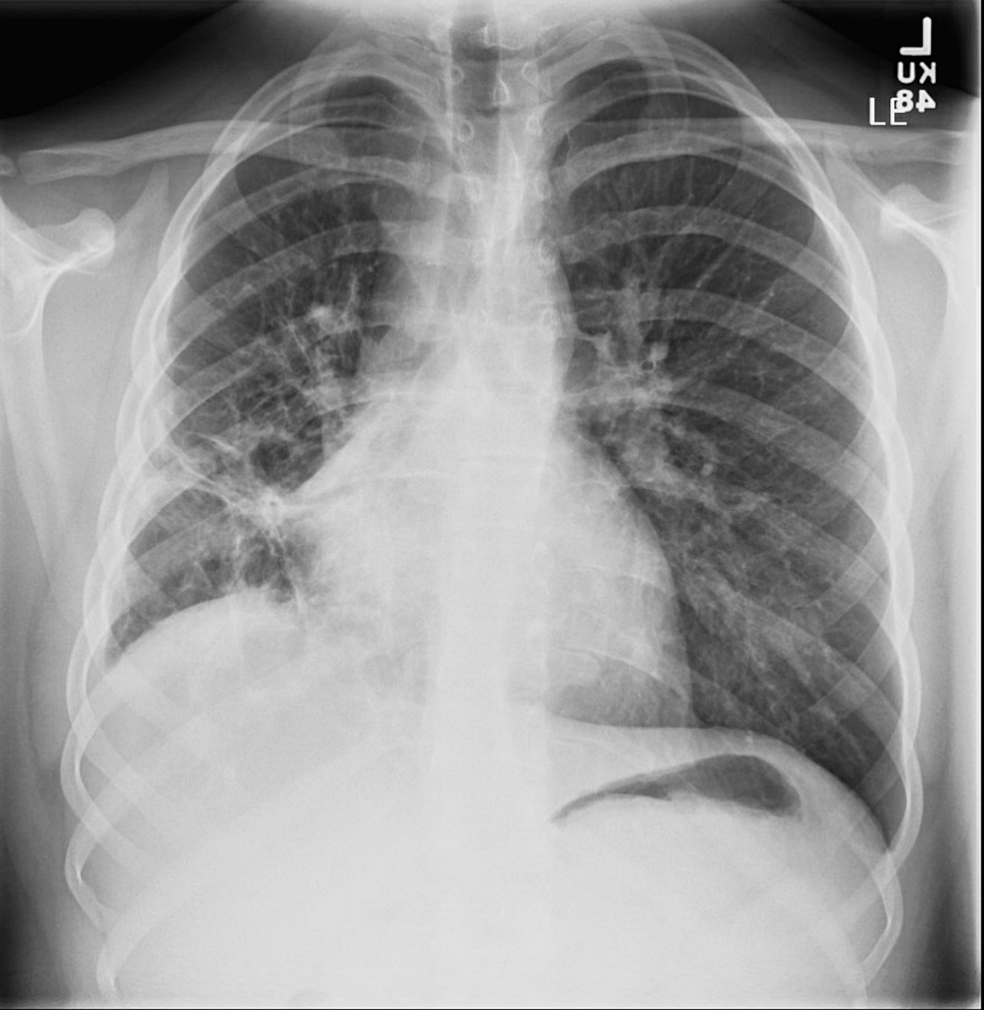
Chest X-rays after four months of treatment Interval improvement of right lower lobe patchy opacity with residual atelectasis in the right middle and lower lobes is observed. There was right pleural thickening, but no effusion was seen.

At that time, the liposomal amphotericin B was switched to oral itraconazole. He continued improving, and he was discharged home after 40 days in the hospital with plans to complete 6-12 months of anti-fungal therapy. The final results of BAL cultures revealed negative bacterial and mycobacterial cultures and a positive fungal culture with a heavy growth of *Blastomyces gilchristii*, confirming the diagnosis of blastomycosis. The patient has been followed as an outpatient by pediatric infectious diseases. He continues improving clinically with excellent weight gain, no residual symptoms, and significant improvement in CXR after two and four months of treatment (Figures 6, 7).

## Discussion

Blastomycosis is mostly caused by *Blastomyces dermatitidis* and *B. gilchristii*, which are genetically different fungi. They cannot be differentiated by routine clinical identification, but they have similar clinical manifestations. They are thermally dimorphic fungi that grow as mold in the environment and as single, thick-walled, and broad-based budding yeast at warmer temperatures, such as the body of humans and other mammals [1,2].

Blastomycosis is endemic in certain parts of the United States including areas surrounding the Mississippi and Ohio River valleys, some southeastern states, and areas bordering the Great Lakes [3]. About 75% of nationally reported cases in 2019 occurred in Minnesota and Wisconsin. However, blastomycosis is only reportable in five states (Arkansas, Louisiana, Michigan, Minnesota, and Wisconsin) [2]. Although the exact numbers are not known, autochthonous blastomycosis appears to be rare in Florida [2]. Therefore, to even suspect blastomycosis, a history of travel to the parts of the United States where blastomycosis is endemic becomes critical.

Blastomycosis is acquired by inhalation of the organism [1,4]. Infection is associated with outdoor occupation and recreation, especially when associated with environmental disruption [1,3]. The incubation period can be two weeks to three months (median: 45 days), and patients often present after days or weeks of mild symptoms [2].

Pulmonary disease is the most common clinical presentation occurring in 79-90% of cases, and the spectrum ranges from mild to severe pneumonia [1,2,4]. In a pediatric series, patients with pulmonary disease had more systemic signs and symptoms such as fever, decreased oral intake, and more elevated markers of inflammation (e.g., C-reactive protein) than children with extrapulmonary disease [4]. In a small percentage of patients, the pulmonary infection can progress rapidly to acute respiratory distress syndrome and can be fatal [3,4]. The initial diagnosis is often CAP because the radiographic findings are similar with consolidation being the most common. However, other associated findings that are not as common in CAP are observed in two-thirds of pediatric patients with pulmonary blastomycosis. These include pulmonary nodules (very often bilateral), reticulonodular opacifications, lymphadenopathy, and large pleural effusion. Additionally, multiple small cavities are found in more than 30% of children, and they are best seen on chest CT [5]. As in our case, it is not unusual that patients receive several courses of antibiotics before the diagnosis of blastomycosis is made. A high index of suspicion is the key to making the diagnosis of blastomycosis, and an epidemiologic link is critical. Even in endemic areas, the diagnosis is often delayed especially in children, and this may lead to worse outcomes [4,6,7].

It is estimated that close to 50% of infected persons are asymptomatic [3]. Extrapulmonary blastomycosis in the form of dermatologic, skeletal, genitourinary, neurologic, or disseminated disease is seen in 25-40% of symptomatic cases [1,3]. Central nervous system involvement (either isolated or as part of disseminated disease) is rare and occurs more often in immunocompromised patients [1,4].

Although the gold standard for diagnosis of blastomycosis is culture, it can take one to five weeks to yield results [1,2]. Visualizing the characteristic appearance of the organism on cytology or histopathology is enough to initiate empiric antifungal therapy while awaiting the results of the culture [3]. Antigen, antibody, and molecular tests are also available, but their usefulness in clinical practice is limited [1].

All patients diagnosed with blastomycosis should be treated with antifungals, including those who are immunocompetent, because of the high risk of dissemination [1,3]. This is unlike certain other endemic fungal infections such as coccidioidomycosis and histoplasmosis, which may not always need treatment [1]. In cases of pulmonary blastomycosis, for moderately to seriously ill patients requiring hospitalization, the initial treatment is with amphotericin B for at least one to two weeks, followed by 6-12 months of oral itraconazole. Mild to moderate cases of blastomycosis may be treated outpatient with itraconazole for 6-12 months [1,3].

This case illustrates the importance of history in the evaluation of patients. History of sick contacts, contact with animals, pets, or, otherwise, exposure to individuals with certain diseases and travel are elements that can be critical in directing the differential diagnosis and guiding the workup. The questions about travel history need to be very specific in regard to the place, duration, activities, exposures, and timing in regard to the beginning of symptoms [8]. In this case, the parents and patient did not consider a camping trip to Minnesota and North Carolina as travel and did not report these trips despite being questioned multiple times about travel. This patient lives in Florida, where blastomycosis is not endemic. However, he had traveled to endemic areas. Knowing our patient’s history of travel would have elevated blastomycosis on the differential diagnosis. This would have triggered targeted testing with BAL, resulting in an earlier diagnosis, appropriate treatment, and better outcomes [7].

## Conclusions

This case illustrates the importance of history taking with specific questions, revisiting the history, and always considering other differentials when a clinical course deviates too much from expected. History taking is a critical aspect of the diagnosis of any disease, but certain aspects of history are even more important when diagnosing infectious conditions. Questions about travel history need to be very specific. Parents may not consider non-vacation or family trips as travel. They may also interpret “travel” as trips outside the United States. For blastomycosis, as well as for other infections with specific endemic distribution, the diagnosis can be delayed if the epidemiologic link is not established. Early diagnosis of blastomycosis would improve patients’ morbidity and mortality outcomes.
